# Clinical, laboratory, and radiological features of community-acquired pneumonia due to *Chlamydia psittaci* and *Legionella pneumophila* confirmed using next-generation sequencing

**DOI:** 10.1080/07853890.2026.2627122

**Published:** 2026-02-12

**Authors:** Ran Cheng, Zhonghua Deng, Fei Lin, Biying Zhang, Jingjin Liang, Ming Lu

**Affiliations:** ^a^ Department of Infectious Diseases, Peking University Third Hospital, Beijing, China; ^b^ Department of Respiratory and Critical Care Medicine, Peking University Third Hospital, Beijing, China

**Keywords:** Chlamydia psittaci pneumonia, community-acquired pneumonia, Legionella pneumophila pneumonia, next-generation sequencing

## Abstract

**Background and objective:**

*Chlamydia psittaci* and *Legionella pneumophila* are common atypical pathogens that cause severe community-acquired pneumonia (CAP). This study aimed to compare the clinical features and outcomes of *Chlamydia psittaci* pneumonia (CPP) and *Legionella pneumophila* pneumonia (LPP) identified using next-generation sequencing (NGS) for accurate identification.

**Methods:**

This retrospective study included 68 patients with CPP and 42 patients with LPP. All cases were confirmed by metagenomic or targeted next-generation sequencing (mNGS/tNGS) of bronchoalveolar lavage fluid, serum, or sputum samples.

**Results:**

Patients with LPP had a higher prevalence of diabetes and were predominantly male. Poultry contact was common in CPP (64.7% vs. 14.3%), whereas recent travel was associated with LPP (47.6% vs. 2.9%). LPP presented with increased extrapulmonary symptoms. Inflammatory marker levels were higher in LPP, including leukocytosis, neutrophilia, C-reactive protein, and procalcitonin (all *p* < 0.05). Organ dysfunction was more frequent in LPP, with higher creatinine levels. Patients with LPP had more severe hypoxemia, required more respiratory support, and had higher intensive care admission rates. Targeted therapy guided by NGS was effective, with no significant differences in mortality or hospital stay between the two groups.

**Conclusion:**

LPP demonstrated greater initial clinical and laboratory severity compared to CPP. Under NGS-guided targeted therapy, both groups achieved comparable outcomes. The observational finding that both pathogens respond to azithromycin and cause severe disease when left undetected underscore the value of guideline-recommended β-lactams/macrolide combination therapy in CAP settings, particularly where these intracellular pathogens remain undiagnosed without NGS.

## Introduction

Community-acquired pneumonia (CAP) is a major global health burden, with lower respiratory tract CAP causing an estimated 2.5 million deaths annually [1]. Among the diverse etiological agents of CAP, atypical causal pathogens have garnered increasing attention due to their diagnostic challenges and potential to cause severe disease [2]. The most common atypical pneumonia pathogens are *Chlamydia pneumoniae*, *Mycoplasma pneumoniae*, Legionella, *Chlamydia psittaci* (psittacosis), and *Coxiella burnetii* (Q fever) [3]. *Legionella pneumophila*, a Gram-negative facultative intracellular bacterium ubiquitously found in natural and artificial aquatic environments worldwide, is the causative agent of Legionnaires’ disease (LD) [4–6]. *Chlamydia psittaci* is an obligate intracellular bacterium that causes psittacosis in humans, following transmission from avian species, which serve as its primary reservoir [7]. Both pathogen cause diseases characterized by prominent extrapulmonary manifestations and a highly variable, nonspecific clinical symptom spectrum, including high fever, muscle and joint pain, nausea, and vomiting, as well as severe symptoms, including respiratory distress, respiratory failure, and multi-organ failure, which can lead to death [8,9]. Recent epidemiological studies have highlighted the considerable impact of *Legionella pneumophila* and *Chlamydia psittaci* in the context of CAP. A multicenter prospective study conducted across 17 hospitals in China revealed that *L. pneumophila* accounted for 11.3% of severe CAP cases, while *C. psittaci* was responsible for 6.8% of cases [10]. These pathogens are particularly concerning as they can cause severe atypical pneumonia that is often unresponsive to standard β-lactam therapy, leading to delayed treatment and increased risk of complications.

Given that *C. psittaci* and *L. pneumophila* detection are not included in routine microbiological testing, CPP and LPP are often underreported, misdiagnosed, or inadequately identified. This diagnostic uncertainty frequently results in empirical broad-spectrum antibiotic therapy being administered, with studies indicating that 69.8–80.9% of patients receive two or more antibiotics simultaneously before definitive diagnosis, contributing to antimicrobial resistance and unnecessary drug exposure [11]. Systematic head-to-head comparisons based on definitive pathogen identification remain scarce.

Next-generation sequencing (NGS) enables unbiased, sequence-based identification directly from clinical specimens, achieving definitive diagnosis independent of culture or antibiotic exposure [12]. Our study leveraged this technology to conduct the largest comparative analysis of CPP versus LPP to date. Our objective was to compare epidemiological patterns, clinical characteristics, laboratory results, radiologic imaging, and, more importantly, treatments and prognosis between CPP and LPP cases identified using NGS.

## 2. Methods

### 2.1. Study design

We conducted a retrospective, single-center observational study between January 1, 2021, and September 30, 2025, at Peking University Third Hospital, which included patients with CAP caused by *C. psittaci* and *L. pneumophila*. Clinical data, including present and previous medical history, ongoing medication, radiology findings, and laboratory findings, were obtained from electronic medical records. Respiratory support categories were defined by their level of invasiveness: 1) No support: No oxygen therapy or conventional low-flow nasal cannula ≤2 L/min; 2. Conventional oxygen therapy: Face mask or nasal prongs delivering >2 L/min; 3. High-flow nasal cannula (HFNC): Heated humidified system delivering ≥ 30 L/min flow with FiO_2_ titration; 4. Non-invasive ventilation (NIV): Bi-level positive airway pressure (BiPAP) or continuous positive airway pressure (CPAP) *via* face/nasal mask; 5. Invasive mechanical ventilation (IMV): Endotracheal intubation or tracheostomy with positive pressure ventilation. All patients were classified based on the highest level of support received within 72 h of admission. The patient inclusion criteria were as follows: age ≥18 years; diagnosis of CAP according to current guidelines [13]; and positive metagenomic next-generation sequencing (mNGS) or targeted next-generation sequencing (tNGS) results from bronchoalveolar lavage fluid (BALF), serum, or sputum samples. The exclusion criteria were as follows: lack of NGS-based diagnostic evidence and missing medical records.

### 2.2. Ethical considerations

The study protocol conformed to the ethical guidelines of the Declaration of Helsinki and was approved by the Institutional Review Board of Peking University Third Hospital, Beijing, China (approval no. 20250641). Written informed consent was waived due to the anonymized retrospective nature of the analysis. Researchers were blinded to the data they were analyzing.

### 2.3. Statistical analysis

Continuous variables were expressed as the mean ± standard deviation and compared using Student’s *t* test when normally distributed, whereas non-normally distributed data were expressed as the median (interquartile range) and tested using the Mann–Whitney *U* test. Categorical variables were presented as frequencies and percentages and compared using the chi-square or Fisher’s exact test. Statistical analyses were performed using SPSS software (version 22.0; IBM Corp., Armonk, NY, USA). A *p* value < 0.05 was considered statistically significant.

### 2.4. NGS diagnostics protocol

The specimens were sent to the Department of Clinical Laboratory of Peking University Third Hospital for pathogenic mNGS (PACEseq, Hugobiotech, Beijing, China) and tNGS (DRseq, Hugobiotech, Beijing, China) detection.

For the detected bacteria (Mycobacterium excluded), fungi (Cryptococcus excluded), and parasites, the positive criteria for the mNGS results were as follows: (1) genome coverage of the unique reads mapped to this microorganism ranked top10 of the same kind of microbes and the microorganism was not detected in the NTC; or (2) RPM-r (RPM_sample_/RPM_NTC)_ was > 10 (RPM_NTC_ ≠ 0).

For the tNGS results, microbial identification was performed based on three key metrics: RPM, RPM ratio (RPM-r), and detected primer ratio (number of detected primer pairs/total designed primer pairs). A positive result was confirmed when the following criteria were met: RPM of targeted pathogens in test samples exceeded 100, RPM-*r* > 5 (RPM_NTC_ ≠ 0); and detected primer ratio > 0.1.

### 2.5. Handling of co-detections and contaminants

Patients with NGS exhibiting co-detection of other bacterial or fungal pathogens at admission were sequentially evaluated by two clinicians. Cases were excluded if co-detections were deemed clinically significant. Contaminants and low-level colonizers identified at low read depths (<5 reads) were disregarded as per our institutional NGS interpretation protocol.

## 3. Results

### 3.1. Comparison of demographic and clinical characteristics

**Figure 1.**
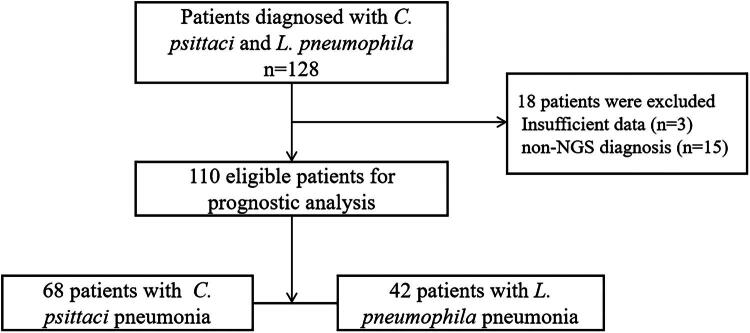
Flowchart of the study.

In this study, 110 patients were included in the final analysis (Figure 1): 68 with CPP and 42 with LPP, all confirmed by mNGS or tNGS. The demographic, clinical, and radiological characteristics of the patients are summarized in Table 1. Among these patients, one with CPP was pregnant. Two patients in the CPP group died from the infection, whereas the remaining patients recovered and were discharged after appropriate treatment.

**Table 1.** Comparison of demographic and clinical characteristics between patients with *C. psittaci* and *L. pneumophila.*

Variable	Total (*n* = 110)	CPP (*n* = 68)	LPP (*n* = 42)	*p* value
Age, median (IQR), years	59.27 ± 14.1	59.19 ± 13.6	59.4 ± 15.04	0.940
Age ≥ 65 years	44 (40.0)	29 (42.6)	15 (35.7)	0.471
Male, *n* (%)	62 (56.4)	33 (48.5)	29 (69.0)	0.035*
Smoking, *n* (%)	34 (31.1)	17 (25.0)	17 (40.5)	0.088
Drinking, *n* (%)	36 (32.7)	19 (29.2)	17 (40.5)	0.173
Travel history, *n* (%)	22 (20.0)	2 (2.9)	20 (47.6)	0.000*
History of poultry exposure, *n* (%)	50 (45.5)	44 (64.7)	6 (14.3)	0.000*
**Comorbidities,** *n* (%)				
Hypertension	52 (47.3)	28 (41.7)	24 (57.1)	0.103
Diabetes	21 (19.1)	9 (13.2)	12 (28.6)	0.047*
Heart disease	11 (10.0)	5 (7.4)	6 (14.3)	0.239
Immunosuppressive disease	9 (8.2)	5 (7.4)	4 (9.5)	0.729
Chronic lung disease	5 (4.5)	2 (2.9)	3 (7.1)	0.368
Chronic kidney diseases	6 (5.5)	2 (2.9)	4 (9.5)	0.200
Cerebrovascular diseases	13 (11.8)	10 (14.7)	3 (7.1)	0.363
Malignancy	7 (6.4)	5 (7.4)	2 (4.8)	0.706
**Vital signs, mean ± SD**				
PaO_2_	68.17 ± 11.57	71.82 ± 12.41	62.92 ± 7.77	0.000*
Temperature, °C	39.09 ± 3.30	38.95 ± 4.15	39.32 ± 0.79	0.570
**Symptom,** *n* (%)				
Fever	110 (100)	68 (100)	42 (100)	1.00
Cough	61 (55.5)	38 (55.9)	23 (54.8)	0.909
Sputum	32 (29.1)	15 (22.1)	17 (40.5)	0.039*
Dyspnea	16 (14.5)	11 (16.2)	5 (11.9)	0.557
Headache	45 (40.9)	29 (42.6)	16 (38.1)	0.637
Dizziness	36 (32.7)	24 (35.3)	12 (28.6)	0.465
Delirium	17 (15.5)	9 (13.2)	8 (19.0)	0.413
Vomit/nausea	24 (21.8)	17 (25.0)	7 (16.7)	0.304
Diarrhea/abdominal pain	13 (11.8)	4 (5.9)	9 (21.4)	0.030*
Urinary system symptoms	11 (10.0)	2 (2.9)	9 (21.4)	0.003*

No statistically significant differences were observed between the two groups in age or comorbidities, including hypertension, heart disease, immunosuppressive conditions, chronic lung disease, chronic kidney disease, malignancy, and cerebrovascular disease. However, patients with LPP exhibited a significantly higher prevalence of diabetes mellitus compared with those with CPP (28.6% vs. 13.2%, *p* = 0.047). The LPP group also had a higher proportion of male patients (29/42, 69.0%) than the CPP group (33/68, 48.5%). A marked contrast was observed in exposure history: direct contact with domestic poultry was substantially more common among patients with CPP, whereas recent travel history was far more frequent in patients with LPP (Table 1).

Fever was present in all the enrolled patients, as well as cough, headache, and dizziness (Table 1). Patients with LPP showed a markedly higher frequency of extrapulmonary symptoms, particularly gastrointestinal and urinary tract manifestations. No significant differences were observed between the two groups in the presence of fever, cough, dyspnea, headache, dizziness, delirium, or nausea/vomiting.

### 3.2. Laboratory and imaging findings at admission

Laboratory findings obtained on admission are summarized in Table 2. Both groups exhibited elevated levels of aspartate aminotransferase (AST), alanine aminotransferase (ALT), lactate dehydrogenase (LDH), d-dimer, and creatine kinase (CK), as well as lymphopenia, hypoalbuminemia, and hyponatremia. However, LPP cases showed more pronounced laboratory derangements, including significantly greater leukocyte and neutrophil counts, and higher serum creatinine (Scr), C-reactive protein (CRP), and procalcitonin (PCT) concentrations (all *p* < 0.05 vs. CPP).

**Table 2.** Laboratory and radiographic characteristics at admission in patients with *C. psittaci* and *L. pneumophila.*

Laboratory findings	Total (*n* = 110)	CPP (*n* = 68)	LPP (*n* = 42)	*p* value
**Blood routine, median (IQR)**				
White blood cell count, ×10^9^/L	8.09 (5.09)	6.92 (3.85)	10.72 (4.88)	0.000*
Neutrophil count, ×10^9^/L	6.87 (4.88)	5.5 (3.27)	9.45 (4.13)	0.000*
Lymphocyte count, ×10^9^/L	0.74 (0.56)	0.77 (0.59)	0.74 (0.50)	0.627
Hemoglobin, g/L	130.5 (22.0)	128 (18.75)	134 (28.5)	0.236
Platelet count, ×10^9^/L	184.5 (78.25)	183.5 (85)	187 (73.75)	0.973
**Blood biochemistry, median (IQR)**				
Aspartate aminotransferase, U/L	40.1 (47.58)	46.5 (56.88)	31.15 (36.55)	0.294
Alanine aminotransferase, U/L	50 (58.5)	53 (58.02)	42.15 (66.55)	0.481
Lactate dehydrogenase, U/L	305 (173)	309 (199.5)	299.5 (136.5)	0.926
Total bilirubin, μmol/L	13.75 (8.85)	12.8 (7.15)	14.8 (11.68)	0.169
Albumin, g/L	36.7 (8.3)	36.6 (7.10)	37.35 (9.55)	0.791
Serum sodium, mmol/L	134.5 (6.73)	134.5 (6.15)	134.65 (8.78)	0.866
Creatinine, μmol/L	82.5 (41.25)	73 (34.75)	94 (53)	0.005*
Creatine kinase, U/L	125 (460.25)	116 (306.25)	130 (530.25)	0.710
**Inflammatory mediators, mean ± SD**				
C-reactive protein, mg/L	186.48 ± 73.07	167.73 ± 68.14	217.73 ± 71.05	0.001*
**Bacterial infection mediators, median (IQR)**				
Procalcitonin, μg/L	0.50 (1.71)	0.37 (1.00)	0.73 (2.99)	0.003*
**Blood coagulation, mean ± SD or median (IQR)**				
d-dimer, μg/mL	0.55 (0.79)	0.48 (0.71)	0.63 (0.81)	0.265
**Radiographic characteristics**				
Lesions in isolated left lung, *n* (%)	33 (30.0)	24 (35.3)	9 (21.4)	0.123
Lesions in isolated right lung, *n* (%)	45 (40.9)	27 (39.7)	18 (42.9)	0.744
Lesions in bilateral lung, *n* (%)	32 (29.1)	17 (25)	15 (35.7)	0.229
**Image changes computed tomography scans**				
Ground-glass opacity, *n* (%)	60 (54.5)	32 (47.1)	28 (66.7)	0.045*
Consolidation, *n* (%)	94 (85.5)	57 (83.8)	37 (88.1)	0.537
Pleural effusion, *n* (%)	31 (28.2)	18 (26.5)	13 (31.0)	0.612

High-resolution thoracic CT showed consolidation as the dominant pattern in both groups, followed by ground-glass opacities (GGO) and pleural effusions (Table 2). Apart from the higher frequency of GGO in the LPP group (66.7% vs. 47.1%, *p* = 0.037), no significant between-group differences were observed in anatomical distribution or in the prevalence of consolidation and pleural effusion (all *p* > 0.05).

### 3.3. Pathogen detection and diagnostic results

Among patients with CPP or LPP, the majority of positive NGS results were derived from BALF samples (61.8% and 61.9%, respectively). When NGS was used as the reference, conventional tests for *L. pneumophila* showed low concordance: only 13 of 42 NGS-positive cases were urinary Legionella antigen-positive, 6 of 42 were IgM-seropositive, and routine cultures yielded no isolates.

### 3.4. Treatment and clinical outcomes

All patients received empirical antibiotic therapy prior to etiological confirmation. All patients who were initially treated with a regimen containing β-lactams had these agents discontinued upon confirmation of LPP and CPP by NGS.

Among 42 patients with LPP, 15 received empirical β-lactam monotherapy. Twenty-three exhibited inadequate responses to the initially administered empirical antibiotics, prompting regimen adjustment to fluoroquinolones, azithromycin, or combination therapy following pathogen identification. Overall, 40 patients (95.2%) exhibited improved infection and were discharged successfully. The remaining two patients (4.8%) who showed clinical improvement were transferred to specialized nursing facilities for further management.

Prior to etiological confirmation, the initial empirical antibiotic regimens varied among the 68 patients with confirmed CPP. In the CPP cohort (*n* = 68), 20 received empirical fluoroquinolones, 26 β-lactam monotherapy, and 8 β-lactam-fluoroquinolone combined therapy, none of which constituted first-line therapy for CPP. Fifty-one showed suboptimal responses to initial therapy and were switched to azithromycin, tetracyclines, or combination regimens after CPP was confirmed. Ultimately, 66 of 68 patients (97.1%) achieved clinical recovery and were discharged. Unfortunately, two patients (2.9%) died from disease progression despite receiving targeted antimicrobial therapy.

Patients with LPP exhibited significantly more severe impairment in gas exchange at presentation than those with CPP, as evidenced by a significantly lower partial pressure of arterial oxygen (Table 1). This greater degree of hypoxemia directly translated into higher dependence on respiratory support in the LPP cohort (Table 3). The proportion of patients who did not require respiratory support was substantially lower in the LPP group than in the CPP group. Consequently, conventional oxygen therapy and high-flow nasal cannula were used significantly more in patients with LPP. In contrast, the need for advanced ventilatory support did not differ significantly between the two groups. The rates of non-invasive mechanical ventilation (NIV) and invasive mechanical ventilation (IMV) were comparable between the two groups. The rate of intensive care unit (ICU) admission was significantly higher among patients with LPP, reflecting their initial disease severity. However, despite the differences in initial severity and ICU admission, the overall length of hospital stays did not differ significantly between the two patient populations. Patients with CPP required continuous renal replacement therapy significantly less often than those with LPP (2.9% vs. 14.3%, *p* = 0.041). However, the frequency of use of glucocorticoids was similar between the groups (*p* > 0.05).

**Table 3.** Treatments and prognosis of patients with *C. psittaci* and *L. pneumophila.*

Variable	Total (*n* = 110)	CPP (*n* = 68)	LPP (*n* = 42)	*p* value
**Diagnostic strategy,** *n* (%)				
Peripheral blood	10 (9.1)	6 (8.8)	4 (9.5)	0.938
Sputum	34 (30.9)	22 (32.3)	12 (28.6)	0.671
Bronchoalveolar lavage fluid	68 (61.8)	42 (61.8)	26 (61.9)	0.981
**Respiratory support mode,** *n* (%)				
No respiratory support	23 (20.9)	22 (32.4)	1 (2.4)	<0.001*
Conventional oxygen therapy	65 (59.1)	34 (50.0)	31 (73.8)	0.003*
High-flow nasal cannula	6 (5.5)	1 (1.5)	5 (11.9)	0.041*
Non-invasive mechanical ventilation	8 (7.3)	6 (8.8)	2 (4.8)	0.252
Invasive mechanical ventilation	8 (7.3)	5 (7.4)	3 (7.1)	0.721
**Other supportive treatments,** *n* (%)				
Glucocorticoids	10 (9.1)	7 (10.3)	3 (7.1)	0.741
Continuous renal replacement therapy	8 (7.3)	2 (2.9)	6 (14.3)	0.041*
**Empirical antibiotic therapies before diagnosis,** *n* (%)				
Quinolones (moxifloxacin, levofloxacin, and nemonoxacin)	29 (26.4)	20 (29.4)	9 (21.4)	0.356
Tetracyclines (doxycycline, minocycline, and tigecycline)	1 (0.9)	1 (1.5)	0	1.000
Azithromycin	3 (2.7)	3 (4.4)	0	0.285
Quinolones + tetracyclines	2 (2.9)	1 (1.5)	1 (2.4)	1.000
Azithromycin + tetracyclines	2 (2.9)	1 (1.5)	1 (2.4)	1.000
Azithromycin + quinolones	1 (0.9)	0	1 (2.4)	0.382
β-lactam monotherapy	41 (37.3)	26 (38.2)	15 (35.7)	0.790
β-lactam + quinolones	16 (14.5)	8 (11.8)	8 (19.0)	0.293
β-lactam + azithromycin	8 (7.3)	5 (7.4)	3 (7.1)	1.000
β-lactam + tetracyclines	7 (6.4)	3 (4.4)	4 (9.5)	0.424
**Pathogen-targeted antibiotic therapies after diagnosis by NGS,** *n* (%)				
Quinolones (moxifloxacin, levofloxacin, and nemonoxacin)	16 (14.5)	3 (4.4)	13 (31.0)	0.000*
Tetracyclines (doxycycline, minocycline, and tigecycline)	31 (28.2)	29 (42.6)	2 (4.8)	0.000*
Azithromycin	35 (31.8)	20 (29.4)	15 (35.7)	0.491
Quinolones + tetracyclines	6 (5.5)	6 (8.8)	0	0.081
Azithromycin + tetracyclines	10 (9.1)	10 (14.7)	0	0.005*
Quinolones + azithromycin	12 (10.9)	0	12 (28.6)	0.000*
**Clinical outcomes,** *n* (%)				
Intensive care unit admission	26 (23.6)	10 (14.7)	16 (38.1)	0.010*
Hospital death	2 (1.8)	2 (2.9)	0	0.521
Time from illness onset to hospital admission	6 (3)	6 (3)	5 (3.25)	0.212
Hospital stay	7 (6)	6.5 (6)	7 (9)	0.157

## 4. Discussion

Our comparative study delineated significant distinctions and similarities in the demographic, clinical, laboratory, and radiological profiles of CAP caused by *C. psittaci* and *L. pneumophila*. Although both pathogens can cause severe disease, our findings consistently indicated that *L. pneumophila* infection presents with greater overall illness severity at admission, as reflected by more pronounced laboratory inflammatory marker levels, a higher incidence of extrapulmonary manifestations, and increased requirements for supplemental oxygen and ICU care.

### 4.1. Epidemiological and host-derived biomarkers of severe disease

A higher number of males in the LPP group (69%) has been reported previously and may reflect occupational or behavioral exposure patterns (e.g. hotel stays or spa use) rather than a genuine biological predisposition [14]. The 2.2-fold higher rate of diabetes in LPP aligns with the findings of population-based studies that have identified diabetes as an independent risk factor for LD, probably through impaired macrophage bactericidal activity and heightened susceptibility to hyperinflammatory cascades [15]. Conversely, the strong poultry-exposure association in CPP (64.7%) reflects the zoonotic reservoir of *C. psittaci* and reinforces the dictum that a history of exposure to poultry is a strong indicator of CPP [16]. The low prevalence of recent travel in CPP (2.9%) contradicts travel-based acquisition of this disease and may guide early empirical treatment choices prior to microbiological confirmation.

### 4.2. Clinical presentation and organ involvement

Clinical presentation of CPP and LPP offers key differentiating characteristics. Although both pathogens typically present as atypical CAP, LPP is distinguished by a significantly higher incidence of gastrointestinal complaints and urinary symptoms, which serve as readily discernible diagnostic cues for clinicians. Although gastrointestinal symptoms are a recognized feature of LD, the prominence of urinary symptoms in our LPP cohort is intriguing and may reflect the systemic effects of the infection or associated complications, warranting further investigation [17]. The laboratory findings further highlighted the heightened inflammatory state in LPP. The significantly higher levels of leukocytosis, neutrophilia, CRP, PCT, and organ injury markers (e.g., CK and Scr) observed in LPP suggest a more vigorous systemic inflammatory response and greater multi-organ involvement than in CPP. This pronounced physiological derangement directly explains the greater need for basic and advanced oxygen support (conventional oxygen and high-flow nasal cannula support) and the higher rate of ICU admission in the LPP group [18]. Importantly, the radiological pattern was largely nondiscriminatory, apart from the higher rate of GGO in LPP, underscoring the necessity of laboratory confirmation rather than reliance on imaging alone.

### 4.3. Diagnostic performance of conventional tests

Among the NGS-confirmed LPP cases, only 31% were urinary antigen-positive, and no isolates were recovered by culture, re-emphasizing the low sensitivity of conventional tests after prior antibiotic exposure or when the infecting strain is a non-serogroup 1 type.

### 4.4. Novel insights from an NGS-confirmed cohort and treatment implications

This direct comparison of NGS-confirmed LPP and CPP within a single-center cohort provides real-world evidence of disease severity and management using rapid molecular diagnostics. Guided by mNGS or tNGS results, we performed targeted escalation or de-escalation of antimicrobial therapy, and both cohorts achieved high recovery rates after pathogen-directed adjustments. These findings support the early integration of mNGS or tNGS into the diagnostic workup of severe CAP, particularly when empirical treatment fails. In this observational setting, clinical outcomes for patients with LPP treated with azithromycin monotherapy were similar to those receiving Fluoroquinolones, consistent with the findings of prior reports [19]. Similarly, for CPP, the outcomes of treatment with Azithromycin alone appeared comparable to those of Tetracycline-based regimens, though this requires validation in larger studies.

### 4.5. Respiratory support and resource utilization

Despite the clear differences in initial disease severity and ICU utilization, the comparable length of hospital stays between the two groups was clear. Additionally, the similar rates of advanced ventilatory support (NIV and IMV) suggest that although LPP causes moderate hypoxemia more frequently, its propensity to progress to the most severe stages of respiratory failure does not significantly differ from that of CPP. In the present cohort, no deaths attributable to LPP occurred, yielding an in-hospital case-fatality rate of 0%, which is substantially lower than the 10–20% rate frequently reported in existing literature [18]. We attribute this favorable outcome to the routine early deployment of NGS, which permitted pathogen identification within 24–48 h of admission and ensured that antimicrobial therapy remained consistently aligned with the Legionella-directed guidelines. These data highlight the pivotal role of rapid molecular diagnostics in reducing LPP-related deaths.

## 5. Limitations

Our study had certain limitations. First, its single-center retrospective design may have introduced selection bias and limited the generalizability of the findings. Second, although substantial for these relatively rare pathogens, the sample size may have been insufficient to detect subtle differences in clinical outcomes. Third, due to sample size constraints and risk of model overfitting, multivariable adjustment for potential confounders (age, sex, diabetes, and chronic lung disease) was not performed. Therefore, the observed associations should be interpreted as unadjusted comparisons that may reflect confounding rather than causal relationships. Finally, although highly sensitive, the reliance on NGS as the primary diagnostic tool did not allow for antimicrobial susceptibility testing.

## 6. Conclusion

In conclusion, this detailed comparative analysis demonstrated that LPP typically presents with greater initial severity than CPP, as evidenced by more marked inflammatory responses and higher oxygen requirements. However, key epidemiological factors and specific clinical features can aid in differentiating these infections. The critical role of rapid molecular diagnostics, such as mNGS or tNGS, is highlighted by the frequent need to adjust therapy following pathogen identification. Azithromycin demonstrated excellent efficacy as a targeted therapy for both LPP and CPP. This finding substantiates the current guideline recommendation of combining a β-lactam with a macrolide for the empirical treatment of hospitalized patients with CAP^13^. This regimen provides critical coverage against these elusive atypical pathogens, thereby improving the outcomes of empirical therapy.

## Data Availability

The data that support the findings of this study are available from the corresponding author, ML (email: luming197954@163.com), upon reasonable request.

## Abbreviations

AST: aspartate aminotransferase
ALT: alanine aminotransferase
CPP: Chlamydia psittaci pneumonia
C. psittaci: Chlamydia psittaci
CAP: Community-acquired pneumonias
ICU: Intensive care unit
LDH: lactate dehydrogenase
LPP: Legionella pneumophila pneumonia
L. pneumophila: Legionella pneumophila
mNGS: Metagenomic next-generation sequencing
tNGS: targeted next-generation sequencing
LD: Legionnaires’ disease
CK: creatine kinase
Scr: serum creatinine
CRP: C-reactive protein
PCT: procalcitonin
CT: computed tomography
GGO: ground-glass opacities
NIV: non-invasive mechanical ventilation
IMV: invasive mechanical ventilation
